# News and Miscellany

**Published:** 1902-01

**Authors:** 


### News and Miscellany.

**The State Association of Health Officers** will meet in Austin on 14th January (inst.)

**Dr. Sims,** of Boerne, Texas, has been appointed State Quarantine Officer at Sabine Pass, vice Tackaberry, removed.

**Dr. C. C. Garritt,** a venerable physician of Calvert, Texas, lost his life in a fire in that city on the night of 29th December (ult.)

**The** New Orleans Board of Health has issued an order requiring property owners to cover all open cisterns with mosquito netting.

**The State Board of Medical Examiners for Texas** will hold its next meeting for examining applicants at Waco, May 13, 1902.

**The oldest physician** in active practice in the United States is said to be Dr. Charles F. Willoglis, of Ohio. Dr. Willoglis is ninety-eight years old.

**An exceedingly** fatal epidemic of measles is said to be prevailing in Alaska. Great numbers of the natives have already died of the disease.

**Attention is called** to the advertisement of Dr. Ed. Cook's Sanatorium at Houston, the "Alcomo." Dr. Cook is an ethical physician, and the Sanatorium will be conducted upon correct principles.

**Dr. J. C. Mayfield,** State Quarantine Officer of Galveston, died at San Antonio December 20th ult. of cirrhosis of the liver. Dr. Norton has been appointed temporarily to take charge of the station.

**To December 1st** 116 deaths from smallpox had occurred in London during the present outbreak of the disease. As usual the mortality was many times as great among the unvaccinated as it was among the vaccinated.

**A cyst** of the vermiform appendix containing six ounces of straw colored fluid is reported in the *New York Medical Journal* by Dr. W. C. Wood of Gloverstown, N. Y. The patient was a young unmarried woman.

**Investigations carried** on at the University of Pennsylvania during the summer under the auspices of the Rockefeller Institute of Preventive Medicine have resulted in the discovery of the specific bacillus of the dysentery we see in this country, thus showing that dysenteries in this and in tropical climates are identical.

---

**According** to *American Medicine* an effort is being made in Paris to have the manufacture of corsets placed under state control because of the organic derangements produced by the wearing of corsets by "young women." The proposed bill provides that no women under thirty shall be allowed to wear a corset under penalty of at least three months imprisonment and a fine of not less than $100.

---

**New Orleans Polyclinic** now in session, 15th year. Closes May 31, 1902.—Physicians will find the Polyclinic an excellent means for posting themselves upon modern progress in all branches of medicine and surgery. The specialties are fully taught, particularly laboratory work. For further information address Dr. Isadore Dyer, Secretary, New Orleans Polyclinic, postoffice box 797, New Orleans, La.

---

**Texas State Medical Association Invited to San Antonio.**—As I go to press notice is received that the West Texas Medical Association last night (January 3) passed a resolution inviting the association to meet in San Antonio in April. See the resolutions by the El Paso Medical Society. As the Confederate Veterans and the C. S. Army and Navy Medical Officers meet in Dallas on the days set for the State Association meeting (April 22-25) the president should change the date to a week earlier or later.

---

### Parke, Davis & Co's. Vaccine.

---

DETROIT, MICH., Dec. 5, 1901.

*F. E. Daniel, M. D., Editor* TEXAS MEDICAL JOURNAL, *Austin, Texas.*

DEAR SIR:—We respectfully ask you to apprise your readers, on the faith of our positive assurance to you, that not one of the recent tetanus fatalities following vaccination at Camden, Atlantic City, Bristol, Brooklyn, Cleveland and St. John, N. B., succeeded the employment of our vaccine virus. In not a single, solitary one of these cases was our vaccine used. We incriminate no one's vaccine, but we propose to assert the truth about our own. If we can prevent it, no physician or pharmacist shall labor under the false impression that a fatality has ever followed, either by coincidence or by cause and effect, the application of vaccine virus or serum bearing our label. Very respectfully yours,

PARKE, DAVIS & CO.

